# Adherence to therapy of patients with adolescent depression

**DOI:** 10.1192/j.eurpsy.2024.316

**Published:** 2024-08-27

**Authors:** V. Kaleda, M. Omelchenko

**Affiliations:** Mental Health Research Center, Moscow, Russian Federation

## Abstract

**Introduction:**

Therapy of adolescent depression is accompanied by a number of difficulties associated with the influence of the age factor, which include the following aspects: social - with the possibility of forming a fear of stigmatization, biological – with low tolerance of psychopharmacological agents due to the immaturity of the functional systems of the body, psychological – with a combination of oppositional behavior of adolescents and their desire to “be like everyone else”. All these factors may lead to a decrease in patients’ compliance in the treatment of adolescent depression and premature refusal to continue treatment, which may provoke a relapse of the disorder.

**Objectives:**

To assess the adherence of adolescent patients with a first depressive episode regarding the continuation of therapy after discharge from the hospital.

**Methods:**

124 patients (average age – 19.4) were examined after discharge from the hospital where they were treated for a depressive episode (according to ICD-10: F32.1, F32.2, F32.38, F32.8). The severity of depression during hospitalization and at discharge was assessed according to the HDRS scale. During hospitalization, 45.9% of patients (n=57) were diagnosed with severe depression (HDRS score of more than 24), 54.1% (n=67) were diagnosed with moderate depression (HDRS score of 17-23) (Zimmerman M. et al. JAD 2013; 150(2):384-8). At discharge, 35.5% of patients (n=44) had moderate depression, 38.7% of patients (n=48) had mild depression (HDRS score of more than 7-16) and only 25.8% of patients (n=32) had no depression (HDRS score of less than 7 points). This indicated the need to continue therapy after discharge. The degree of adherence to therapy and the main reasons for its refusal were analyzed using the Medication Compliance Scale (Lutova N. NIPNI, 2007; 26).

**Results:**

The average duration of treatment continuation in patients with adolescent depression was 7.4±9.6 months. At the same time, 42 patients (33.9%) refused to continue therapy within 30 days after discharge from the hospital. 15 patients (12.1%) turned out to be fully compliant, following the doctor’s prescriptions. The main reasons for refusing therapy were: negative attitude to the fact of receiving therapy and visiting a psychiatrist (n=50, 40.3%), the development of side effects of therapy (n=46, 37.1%), negative attitude of relatives to the continuation of therapy (n=11, 8.9%), and negative attitude to the attending psychiatrist (n=2, 1.6%). In general, formally, the average duration of continuation of therapy coincides with the recommended 6-12 months (Sim K. et al. IGN 2015;19(2) pyv076), however, it is noteworthy that some patients tend to self-cancel therapy without the approval of the attending physician.

**Conclusions:**

The results indicate a low level of adherence to therapy in patients with adolescent depression and require additional measures to improve it.

The work was carried out with the financial support of the RSF grant 22-15-00437.

**Disclosure of Interest:**

None Declared

